# Case Report: First Occurrence of Plasmablastic Lymphoma in Activated Phosphoinositide 3-Kinase δ Syndrome

**DOI:** 10.3389/fimmu.2021.813261

**Published:** 2021-12-21

**Authors:** Zexi Yin, Xin Tian, Runying Zou, Xiangling He, Keke Chen, Chengguang Zhu

**Affiliations:** Department of Hematology and Oncology, Children’s Medical Center, Hunan Provincial People’s Hospital/The First Affiliated Hospital of Hunan Normal University, Changsha, China

**Keywords:** children, PIK3CD gene, activated phosphoinositide 3-kinase δ syndrome, EBV, plasmablastic lymphoma

## Abstract

Activated phosphoinositide 3-kinase δ syndrome (APDS) is an autosomal dominant primary immunodeficiency caused by acquired gene function mutation (GOF). APDS has a variety of clinical phenotypes, particularly recurrent respiratory infections and lymphoproliferation. Here we report a pediatric patient with APDS who presented with recurrent respiratory infections, lymphoproliferation, hepatosplenomegaly, bronchoscopy suggesting numerous nodular protrusions in the airways and a decrease in both T and B lymphocytes, and progression to plasmablastic lymphoma (PBL) after 1 year. Whole exome sequencing revealed a heterozygous mutation in the PIK3CD gene (c.3061 G>A p.E1021K). This is the first reported case of APDS combined with PBL and pediatricians should follow up patients with APDS regularly to be alert for secondary tumours.

## Introduction

Activated phosphoinositide 3-kinase δ syndrome (APDS) is an autosomal dominant primary immunodeficiency. It is caused by PIK3CD gene mutations that encodes PI3K δ catalytic subunit p110δ (APDS1) or regulatory subunit p85α (APDS2) (1). PIK3CD mutations identified include E1021K, N334K, C416R, E525K, G124D, E81K; the most common is E1021K (c.3061G>A) (2).

The phenotypes of APDS are highly variable, ranging from asymptomatic adults to profound immunedeficiency causing early death ornecessitating haematopoietic stem cell transplantation (HSCT) in childhood (3). It usually presents with recurrent respiratory infections, lymphoproliferation, gastrointestinal manifestations, autoimmune disease and an increased risk of malignancy. 49% of patients with APDS1 have severe, persistent or recurrent herpesvirus infections, including EBV, CMV, HSV and VZV infections (4). A high incidence of lymphoma has also been recorded in patients with APDS. The main immunological features of APDS are reduced numbers of CD4+ T and B cells and reduced IgG levels; 58% of patients with normal IgG levels have IgG2 subclass defects; reduced IgA and elevated IgM are common (5, 6).

At present, although a number of combined lymphomas have been reported in patients with APDS, diffuse large B-cell lymphoma is the most common, and nodular sclerosis classical Hodgkin lymphoma, nodar marginal zone lymphoma and lymphoplasmacytic lymphoma have also been reported (7). However, PBL has not been previously reported. Here, we report a case of APDS combined with PBL. The aim of this case report is to draw the attention of pediatricians to the diversity of APDS combined with lymphoma and to explore the feasibility of HSCT in curing APDS combined with PBL.

## Case Presentation

**Figure 1:**
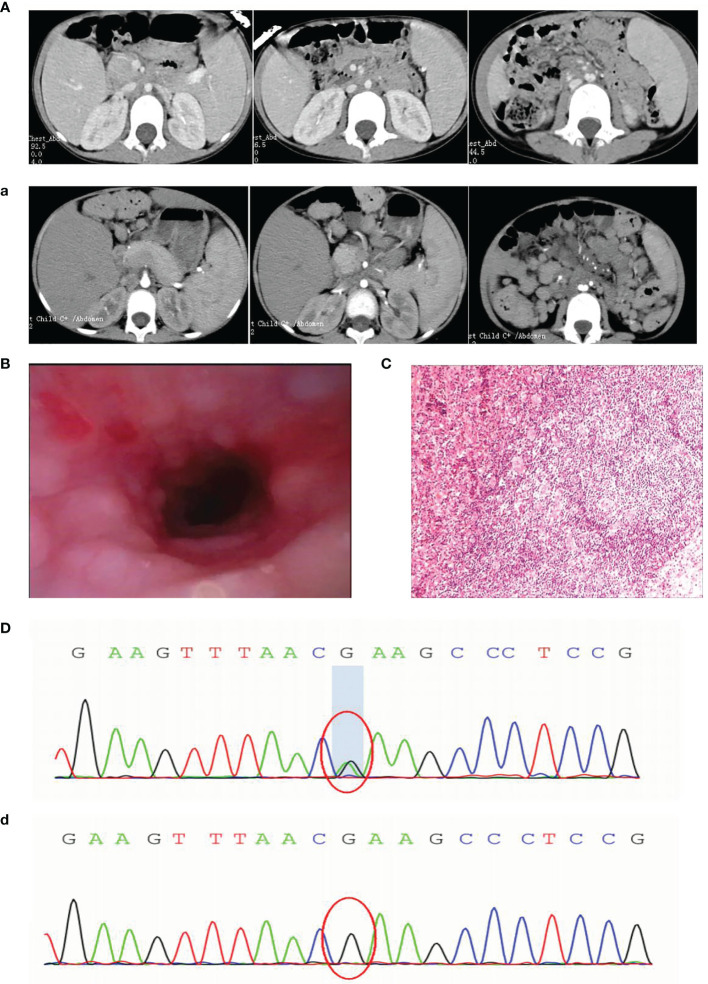
**(A)** First CT: enlarged lymph nodes **(a)** 1 year later CT: extensive lymph node enlargement **(B)** Fibroscopy: numerous nodular protrusions **(C)** Lymph node biopsy: EBV-associated lymphoproliferative lesions (EBER+) **(D)** Sanger sequencing images before transplantation **(d)** Sanger sequencing images after transplantation.

In 2019, a 5-year-old girl was admitted to our hospital with a cough for more than 1 month and coughing up blood with fever for 1 day. Prior to this, she had had recurrent respiratory infections since the age of 6 months. Physical examination on admission revealed visible tonsils, enlarged cervical, axillary and inguinal lymph nodes; dry and wet rales in both lungs; no heart murmur; soft abdomen with hepatosplenomegaly; and no positive neurological signs. Laboratory tests showed no Epstein-Barr virus (EBV) IgM and Cytomegalo virus (CMV) IgM detected on serological EBV antibody screen and CMV antibody screen, but elevated plasma test for EBV-DNA(2.5 hundred copies/ml). Prior to receiving any immunosuppressive treatment, immunologic evaluation was performed and revealed normal serum levels of IgG, IgA and IgM, and a severe T-cell lymphopenia, as well as B-cell lymphopenia, but the percentage of NK-cell was elevated (**Table 1**). Multi-site CTA showed multiple enlarged lymph nodes in the neck, mediastinum, hilum, axilla, retroperitoneum, and groin, multiple lesions in both lungs, and hepatosplenomegaly. (**Figure 1A**). Fibreoptic bronchoscopy revealed a large number of scattered nodular protrusions throughout the trachea and knuckle (**Figure 1B**). Biopsy of the bronchial mucosa showed chronic inflammatory changes in the mucosa and lymphoid hyperplasia, proliferative lesions of lymphoid tissue are not excluded. EBV-associated lymphoid hyperplastic lesions (EBER+) confirmed by cervical lymph node biopsy (**Figure 1C**). In view of the above presentation and characteristics, we advised the family to refine the genetic examination and obtained parental consent. Whole-exome sequencing showed that the patient had a heterozygous mutation (c.3061 G>A p.E1021K) in the PIK3CD gene (**Figure 1D**), and the family genealogy verified that both parents were normal. She was treated with gammaglobulin, ganciclovir and antibacterial drugs; fever and pulmonary symptoms resolved. However, the patient did not return to the hospital for follow-up treatment after discharge.

**Table 1:** Clinical characteristics of the patient.

	First time	1 year later	Reference
EB-VCA-IgG	**180**	**102**	0-20 U/ml
EB-VCA-IgM	<10	<10	0-40 U/ml
EB-EA-IgG	**＞150**	**＞150**	0-40 U/ml
EB-NA-IgG	**＞600**	**91.3**	0-20 U/ml
EB-DNA	**2.50E+02**	**2.50E+02**	<4.0E+02 Copies/ml
**EBV lymphocyte subpopulations(qPCR)**			
CD3+CD4+ cell		Not Detected	
CD3+CD8+ cell		Not Detected	
CD3-CD19+ cell		**1.7E+04**	
CD56+ cell		Not Detected	
**Immunologic**			
Immunoglobulin G	11.1	**3.32**	7.0-16.0 g/L
Immunoglobulin A	1.55	**0.43**	0.7-4.0 g/L
Immunoglobulin M	1.15	**0.17**	0.4-2.3 g/L
IgG 1		**3.04**	4.05-10.11 g/L
IgG 2		**0.44**	1.69-7.86 g/L
IgG 3		**0.09**	0.129-0.789 g/L
IgG 4		**0.00518**	0.013-1.446 g/L
Lym T(CD3+ %)	**47.99**	62.47	59.7-77.6%
Lym T(CD3+ #)	**428**	1810	1578-3707/ul
CD3+CD4+ %	**24.94**	**24.72**	31.1-47.4%
CD3+CD8+ %	17.21	**35.94**	16-26.9%
CD3+CD4+ #	**213**	**714**	870-2144/ul
CD3+CD8+ #	**159**	**1014**	472-1107/ul
Lym B(CD19+ #)	**269**	**258**	434-1274/ul
NK(CD16+CD56+ #)	**890**	**824**	155-565/ul
Lym B(CD19+ %)	**9.58**	**9.06**	12.9-29.2%
NK(CD16+CD56+ %)	**31.86**	**27.86**	4.7-16.2%
**Tumour immunohistochemistry**			
CD3		**Part +**	
CD4		**Part +**	
CD5		**Scattered in +**	
CD8		**Small amount +**	
CD10		**-**	
CD20		**Focal +**	
CD23		**Small amount +**	
CD30		**Scattered in +**	
CD138		**Bulk +**	

In bold values outside the reference range.

At the end of 2020, the girl was readmitted to hospital with enlarged lymph nodes, diarrhea for over 2 months and generalized swelling for 10 days. EBV-DNA remained elevated on admission and immunological evaluation: serum IgG, IgA and IgM were decreased, T and B lymphocytes were decreased and NK cells were elevated (**Table 1**). Multi-site CTA revealed more extensive generalized lymph node enlargement and hepatosplenomegaly than previously (**Figure 1a**). Final cervical lymph node biopsy suggested aggressive lymphoma of the lymph nodes, consistent with plasmablastic lymphoma; IgHV-FR1 (-), IgHV-FR2 (-), IgHV-FR3 (-), IgK-Vk-Jk (+), IgK-Vk-Kde+INTR-Kde (+). The bone marrow was not involved. After obtaining family consent, we gave sirolimus-targeted therapy; and CHOP regimen combined with bortezomib chemotherapy. After four chemotherapy sessions, the child’s symptoms improved significantly and the lymph nodes shrank significantly.

Although the patient’s symptoms improved, complete remission of the disease could not be achieved, so we recommended an allogeneic HSCT. Fortunately, we found a fully compatible HLA donor (10/10) through a Chinese bone marrow bank match, passed the medical examination and obtained the donor’s consent. The pretreatment regimen was busulfan combined with cyclophosphamide and rabbit anti-human thymocyte immunoglobulin, and peripheral blood stem cell transfusion of CD34+ 6.65×10^6^/kg; post-transplantation anti-rejection therapy with cyclosporine combined with morte-macrolimus, neutrophil engraftment (≥0.5×10^9^/kg) at 15 days and platelet engraftment (≥20×10^9^/kg) at 24 days post-transplantation, and chimerism has been maintained at over 95%. Thankfully, this was a successful transplant and the patient was genetically negative at the 1 month post-transplant review (**Figure 1d**). Although hemorrhagic cystitis developed at the time, it was quickly cured with treatment. It is now over 6 months post-transplant and the child has achieved complete remission with disappearance of symptoms, genetic conversion and imaging suggestive of no enlarged lymph nodes and a normal liver and spleen.

## Discussion

We describe a pediatric patient with APDS1 presenting with recurrent respiratory infections, lymphoproliferation, and hepatosplenomegaly. Whole-exome sequencing revealed a mutation in the PIK3CD gene E1021K (c.3061G>A). The mutation was not found in either of the patient parents, suggesting that the mutation was *de novo*. Consistent with APDS, the patient had decreased T and B cells and decreased IgG and IgA, but unlike most cases with increased IgM, her IgM was decreased. It should also be noted that the patient’s immunoglobulins were normal at the time of the initial consultation, which may be related to the intravenous immunoglobulins administered prior to hospitalization. Unfortunately, she also had a combination of lymphoma, an extremely rare type of tumour - PBL.

PBL is a large B-cell lymphoma with a plasma cell phenotype and poor prognosis, first seen in human immunodeficiency virus (HIV)-positive patients with a predilection for the oral cavity; it is noteworthy that EBV infection also causes an abnormal surge in plasma cell levels in the blood and lymph nodes (8). This disease is common in patients with immunodeficiency diseases and post-transplantation (9). Currently, the use of standard regimens to treat offers limited improvement in patient survival, as there is no standard treatment protocol for PBL. Studies have shown that patients with PBL treated with CHOP and CHOP-like regimens have a complete remission rate (CR) of 69%, and a median survival time of 8-15 months when treated with guideline-recommended high-intensity chemotherapy regimens such as DA-EPOCH (10, 11). However, elderly and immunosuppressed patients do not benefit from high-intensity regimens. In recent years, patient prognosis has improved with the development of new drugs such as bortezomib. As reported in a review by Castillo et al. (PMID 29527667) the complete remission rate of PBL after bortezomib combined with EPOCH was over 90% (12).

However, this child had an immunodeficiency (APDS), that may have contributed to the development of PBL, and further damage to the child immune system through strong chemotherapy made it difficult to obtain complete remission of the disease, therefore, immune reconstitution through HSCT may be the best option. Treatment of APDS includes supportive therapies such as prophylactic antibiotics and/or immunoglobulin replacement therapy (IRT). Rapamycin/sirolimus, which directly targets mTOR and inhibits the PI3K pathway downstream, has shown good efficacy in the treatment of lymphoproliferative disorders, and PI3Kδ inhibitors, which interfere with PI3K synthesis and inhibit the PI3K pathway upstream. But the use of mTOR inhibitors and selective PI3K inhibitors in children still requires further follow-up and close observation (13). There is also HSCT. Dimitrova et al. (PMID 34033842) conducted a retrospective analysis of 57 patients with APDS who underwent HSCT and concluded that APDS was reversible by HSCT. In terms of donor selection, there were no statistically significant differences in outcomes based on donor type, although MSD was usually the preferred donor type in practice. Pretreatment is dominated by marrow clearing regimens and reduced intensity regimens, longer detailed follow-up on graft stability, late toxicity, immune reconstitution and phenotypic reversal is still needed to further inform the optimal timing and approach for APDS patients to undergo HSCT (14). This case achieved complete remission by HSCT, but only one case has been reported and there are some limitations.

## Conclusion

APDS is a primary immunodeficiency disease and PBL is often secondary to immunodeficiency. This study is the first to report a case of childhood PBL secondary to APDS. Treatment options such as antibiotics, IRT, targeted drugs and HSCT are available for simple APDS, and patients with PBL can achieve some remission with chemotherapy, but PBL patients with immunodeficiency cannot benefit from intense chemotherapy. The choice of HSCT to cure patients with primary immunodeficiency disease, which can simultaneously achieve an anti-tumour effect during immune reconstitution and result in complete remission of the patient’s tumour, is a win-win option.

## Data Availability Statement

The original contributions presented in the study are included in the article/supplementary material. Further inquiries can be directed to the corresponding author.

## Ethics Statement

The studies involving human participants were reviewed and approved by Medical Ethics Committee of Hunan Provincial People’s Hospital([2021]-77). The patients/participants provided their written informed consent to participate in this study.

## Author Contributions

ZY designed the study and wrote the paper. RZ performed bibliographical research and was medical doctor of the patient. XH performed genetic analysis and reviewed the manuscript. XT performed immunological analysis. KC designed the study and analyzed the data. CZ performed pathology analysis. All authors contributed to the article and approved the submitted version.

## Funding

This work was supported by the Hunan Provincial Science and Technology Department project (2018SK21216). 

## Conflict of Interest

The authors declare that the research was conducted in the absence of any commercial or financial relationships that could be construed as a potential conflict of interest.

## Publisher’s Note

All claims expressed in this article are solely those of the authors and do not necessarily represent those of their affiliated organizations, or those of the publisher, the editors and the reviewers. Any product that may be evaluated in this article, or claim that may be made by its manufacturer, is not guaranteed or endorsed by the publisher.
